# Editorial: Clinical Cases in Cardiovascular Medicine: 2021

**DOI:** 10.3389/fcvm.2022.930230

**Published:** 2022-05-20

**Authors:** Maurizio Acampa, Leonardo Roever

**Affiliations:** ^1^Stroke Unit, Department of Emergency-Urgency and Transplants, Azienda Ospedaliera Universitaria Senese, “Santa Maria alle Scotte” General Hospital, Siena, Italy; ^2^Department of Clinical Research, Federal University of Uberlandia, Uberlândia, Brazil

**Keywords:** myocardial infarction, systemic inflammation, pheocromocytoma, myocardial bridging, extracorporeal shockwave myocardial revascularization, multisystem inflammatory syndrome, Wolff-Parkinson-White syndrome, eosinophilic granulomatosis

In the present Frontiers Research Topic, an international selection of high-quality case reports contributed to advance our understanding of personalized approaches to cardiovascular diagnosis and treatment, beginning with the patient physician communication, to bedside clinical assessment, advanced diagnostic and imaging technologies.

Indeed, these case reports provided insight into the differential diagnosis, decision making, and clinical management of unusual cases, also representing a valuable educational tool.

Several contributions (Li and Liu; Wu et al.; Ye et al.) focused on rare symptoms that can be occur in specific cardiac diseases, suggesting the presence of multiple and complex pathogenic mechanisms (1, 2).

In this view, Ye et al. presented a case of acute myocardial infarction as a rare complication of acute chlorpyrifos poisoning. The complex relationships between poisoning and myocardial infarction are not only represented by the great variety of symptoms, but also by the conflicts of treatments for both conditions. Indeed, atropinization contributes to the control of muscarinic symptoms of chlorpyrifos poisoning, but can also increase the heart rate and myocardial oxygen consumption, which can worse myocardial ischemia.

Wu et al. showed that some pathological conditions can be diagnosed at the onset of rare symptoms, apparently unrelated with the disease as in the case of a hypertensive 59-older patient with covert pheochromocytoma who had a sudden hypotension and shock. These symptoms are rare and apparently inconsistent with pheocromocytoma, but a possible pathogenic explanation can be related to tumor necrosis that leads to a sudden decrease in continuous catecholamine secretion, with subsequent hypotension.

Furthermore, Li and Liu described another atypical case characterized by a usually benign cardiac congenital anatomical variation, the myocardial bridging (MB) of the coronary artery. However, in the case of a 41-year-old man, the association between MB and hypothyroidism contributed to the occurrence of myocardial infarction. In fact, MB initiated the development of coronary atherosclerotic lesions, but hypothyroidism further contributed to the occurrence of myocardial infarction by multiple mechanisms including endothelial dysfunction, increased platelet activation, hypercholesterolemia, increased levels of low-density lipoprotein cholesterol, and hypertriglyceridemia.

Another study by Akbar et al. focused on the efficacy of specific therapies, such as extracorporeal shockwave myocardial revascularization. Akbar et al. presented a case series of four patients with coronary artery bypass grafting-stable angina pectoris who refused surgery and who underwent extracorporeal shockwave myocardial revascularization obtaining an improvement of the ischemic response, functional capacity, and physical component of quality of life.

Systemic inflammation is another pathogenic factor that can interact at multiple levels on the cardiovascular system and many case reports in the present Research Topic pointed out its relevant role. Immuno-inflammatory mechanisms may play a relevant pathogenic role in some cardiac diseases, contributing to development of coronary artery disease (3) and cardiac arrhythmias, modulating both atrial (4, 5) and ventricular substrates (6). The role of inflammatory cytokines has recently become more clear with COVID-19, a systemic inflammatory disease, that can cause myocardial injury (7, 8), with an unexpectedly high prevalence of arrhythmic events (9).

In this respect, Bemtgen et al. described a case of an 18-year-old male patient affected by a multisystem inflammatory syndrome, a novel hyperinflammatory syndrome associated with SARS-CoV-2 infection, where a myocardial biopsy revealed small vessel-associated immune cell infiltrates, without myocardial necrosis, with fast and favorable response to immunomodulatory therapy.

Inflammation can also play an important role in other inflammatory diseases: Cui et al. described an interesting case of eosinophilic granulomatosis with polyangiitis that was manifested as myocardial infarction with non-obstructed coronary arteries. Also in this case, the immunosuppressive therapy led to regression of symptoms with significant clinical resolution.

Alania-Torres et al. described a rare case of patient affected by arrhythmogenic left ventricular cardiomyopathy who developed a myocarditis induced by coronavirus disease 2019 (COVID-19) mRNA vaccine. This case report is particularly interesting from a diagnostic and pathogenic point of view, because both conditions myocarditis and a hot phase of the arrhythmogenic left ventricular cardiomyopathy can have similar ECG, echocardiographic and MRI findings and, moreover, both might be pathophysiologically related.

Yang et al. suggested the further complexity of the relationships between immune modulation and cardiac disease presenting a case of a 33-year-old man with a history of metastatic thymoma treated with sintilimab, who developed grade 3 immune checkpoint inhibitor (ICI)-related myocarditis, complicated with myositis/myasthenia gravis.

Wang et al. described a case of a patient with pneumonia and myocarditis, characterized by the coexistence of Wolff-Parkinson-White (WPW) syndrome and Brugada electrocardiogram (ECG) patterns. Even if this association has already been reported in previous papers, the peculiarity of this case is due to the particular dynamic changes of QRS complex, relating to fever, suggesting the possible modulating role of inflammation on cardiac electrical activity.

In conclusion, the high-quality contributions presented in this Research Topic significantly enriched our knowledge about the field of cardiovascular diseases, shedding light on rare symptoms, complex physiologic and pathogenic mechanisms, that can have relevant implications also for the choice of appropriate treatments in these patients. These studies also provide important suggestions for further investigation in this area.

## Author Contributions

MA and LR contributed to the conception, design, and drafting of the work. All authors contributed to the article and approved the submitted version.

## Conflict of Interest

The authors declare that the research was conducted in the absence of any commercial or financial relationships that could be construed as a potential conflict of interest.

## Publisher's Note

All claims expressed in this article are solely those of the authors and do not necessarily represent those of their affiliated organizations, or those of the publisher, the editors and the reviewers. Any product that may be evaluated in this article, or claim that may be made by its manufacturer, is not guaranteed or endorsed by the publisher.

## References

[1] MacLellan WR Wang Y Lusis AJ. Systems-based approaches to cardiovascular disease. Nat Rev Cardiol. (2012) 9:172–84. 10.1038/nrcardio.2011.208 22231714 PMC3823242

[2] Doran S Arif M Lam S Bayraktar A Turkez H Uhlen M. Multi-omics approaches for revealing the complexity of cardiovascular disease. Brief Bioinform. (2021) 22:bbab061. 10.1093/bib/bbab061 33725119 PMC8425417

[3] Montarello NJ Nguyen MT Wong DTL Nicholls SJ Psaltis PJ. Inflammation in coronary atherosclerosis and its therapeutic implications. Cardiovasc Drugs Ther. (2022) 36:347–62. 10.1007/s10557-020-07106-6 33170943

[4] Lazzerini PE Laghi-Pasini F Acampa M Srivastava U Bertolozzi I Giabbani B. Systemic inflammation rapidly induces reversible atrial electrical remodeling: the role of interleukin-6-mediated changes in connexin expression. J Am Heart Assoc. (2019) 8:e011006. 10.1161/JAHA.118.011006 31423933 PMC6759884

[5] Lazzerini PE Acampa M Cupelli M Gamberucci A Srivastava U Nanni C. Unravelling atrioventricular block risk in inflammatory diseases: systemic inflammation acutely delays atrioventricular conduction via a cytokine-mediated inhibition of connexin-43 expression. J Am Heart Assoc. (2021) 10:e022095. 10.1161/JAHA.121.022095 34713715 PMC8751850

[6] Lazzerini PE Acampa M Laghi-Pasini F Bertolozzi I Finizola F Vanni F. Cardiac arrest risk during acute infections: systemic inflammation directly prolongs QTc interval via cytokine-mediated effects on potassium channel expression. Circ Arrhythm Electrophysiol. (2020) 13:e008627. 10.1161/CIRCEP.120.008627 32654514

[7] Del Prete A Conway F Della Rocca DG Biondi-Zoccai G De Felice F Musto C. COVID-19, acute myocardial injury, and infarction. Card Electrophysiol Clin. (2022) 14:29–39. 10.1016/j.ccep.2021.10.004 35221083 PMC8556597

[8] Aras Júnior R Durães A Roever L Macedo C Aras MG Nascimento L. The impact of COVID-19 on the cardiovascular system. Rev Assoc Med Bras. (2021) 67(Suppl 1):163–16. eCollection 2021. 10.1590/1806-9282.67.Suppl1.20201063 34259776

[9] Lazzerini PE Laghi-Pasini F Boutjdir M Capecchi PL. Inflammatory cytokines and cardiac arrhythmias: the lesson from COVID-19. Nat Rev Immunol. (2022) 2022:1–3. 10.1038/s41577-022-00714-3 35347295 PMC8959266

